# Heterogeneity of resilience and the curvilinear relationship between childhood trauma and resilience among people with schizophrenia

**DOI:** 10.3389/fpsyt.2023.1082000

**Published:** 2023-05-15

**Authors:** Weiliang Wang, Jun Zhang, Xinzhu Zheng, Guohua Li, Yuqiu Zhou

**Affiliations:** ^1^School of Nursing, Xuzhou Medical University, Xuzhou, Jiangsu, China; ^2^School of Nursing, Harbin Medical University, Harbin, Heilongjiang, China; ^3^Heilongjiang Sengong Red Cross General Hospital, Harbin, Heilongjiang, China; ^4^The First Psychiatric Hospital of Harbin, Harbin, Heilongjiang, China; ^5^Chifeng Anding Hospital, Chifeng, China

**Keywords:** schizophrenia, childhood trauma, resilience, curvilinear relationship, stress

## Abstract

**Background:**

As a group with a high incidence of childhood trauma, the differential characteristics of resilience in schizophrenia and its relationship with childhood trauma are still unclear.

**Methods:**

A total of 626 individuals diagnosed with schizophrenia were selected from four psychiatric hospitals in northern China. Childhood trauma and resilience were measured using the Childhood Trauma Questionnaire-short form (CTQ-SF) and Connor–Davidson Resilience Scale (CD-RISC), respectively. Latent profile analysis (LPA) was used to identify the potential classes of resilience. A generalized additive mixed model (GAMM) and restricted cubic spline (RCS) was used to explore and determine the shape of the relationship between childhood trauma and resilience.

**Results:**

Model fits of LPA showed three heterogeneous classes of resilience: moderate resilience levels (*n* = 312; 49.06%); high resilience levels (*n* = 171; 26.89%) and low resilience levels (*n* = 153; 24.06%). Resilience scores fluctuated depending on amount of exposure to childhood trauma. The GAMM results verified the non-linear relationship between resilience and childhood trauma, with an estimated degree of freedom higher than 1 (4.573) and *p* = 0.003. RCS fitted for ordinary least square (OLS) regression models determined a smooth continuous cubic curve of resilience across childhood trauma levels, and the two turning points of the curve line were 41.04 and 54.74 for childhood trauma.

**Discussion:**

Our findings confirm the people-specific differences in the level of resilience in schizophrenia and describe the cubic curvilinear relationship between childhood trauma and resilience, which provides data support for mechanistic research and intervention in related fields.

## Introduction

Individuals with schizophrenia, a severe and chronic mental illness, often deviate from the normal developmental trajectory, resulting in a series of adverse psychological characteristics and functional outcomes during the occurrence and development of the disease. With the application of positive psychology, researchers have paid increasing attention to the role of positive psychological traits in schizophrenia (1, 2). Resilience is one of the important variables in the field of positive psychology, and existing research confirms that resilience has protective and buffering effects between risk factors and multiple clinical outcomes (3). At the heart of resilience is an appropriate stress response and the ability to quickly and effectively recover from stress exposure. During the neurodevelopmental stage, the interaction of genes and the environment has a lasting impact on neural circuits and stress systems, and brain function activation and cognitive reappraisal are also involved in the formation of resilience (4). Existing research suggests that resilience is not just a single psychological concept; it includes a series of biological and social system characteristics (4, 5). Therefore, people-specific characteristics of resilience and their relationship to social systems are of great value. Research has shown that people who experience adversity will have different coping responses and exhibit heterogeneous resilience traits, which are important determinants of recovery from schizophrenia (6, 7). It is therefore necessary to distinguish subgroup clusters of resilience based on a people-centered analysis.

Studies have shown that 47.5–77.5% of individuals with schizophrenia experience varying degrees and types of childhood trauma (8). Childhood trauma, as a detrimental factor in early social development, contributes to and influences individual neurodevelopmental trajectories, leading to a range of adverse outcomes in adulthood. Previous studies have focused on the monotonic relationship between childhood trauma and resilience and its impact on clinical outcomes (1, 3). However, stress events and mental health are not monotonically related. Exposure to moderate stress leads to stronger stress coping ability and better mental health (9–11). Moderate adversity may promote adaptive functioning and resilience to subsequent adversity more than no or high levels of adversity (12–14). According to the stress inoculation hypothesis, a certain degree of childhood trauma experience will increase the inoculation effect of individuals in the face of stress or adversity in adulthood, thereby improving resilience (15). A series of studies have also examined the curvilinear relationship between traumatic experiences and mental health in different populations and contexts (12, 16). However, the non-linear relationship between childhood trauma and resilience in schizophrenia still unclear. Whether there is a quadratic or more complex relationship between childhood trauma and resilience in schizophrenia with a high prevalence of childhood trauma is the question of interest for this study. Therefore, this study aims to (1) explore the heterogeneity of resilience in schizophrenia based on people-center analysis and (2) explore the curvilinear relationship between childhood trauma and resilience.

## Participants and methods

### Subjects

All participants were recruited from four psychiatric hospitals in Chifeng city of the Inner Mongolia autonomous region and Daqing city and Harbin city of Heilongjiang Province in China. The inclusion criteria were (1) International Classification of Diseases, 10th Revision (ICD-10) diagnosis of schizophrenia; (2) clinical stability and absence of aggressive or hostile behavior; (3) age 18 years or older; (4) ability to understand the survey instructions and willingness to provide written informed consent; and (5) sufficient cognitive capacity [a Positive And Negative Symptoms Scale-G12 score less than 4 (17)]. The exclusion criteria were as follows: coexisting intellectual disability, dementia, other severe organic disorders, or drug or alcohol abuse.

### Tools

#### Resilience

Resilience was assessed using the Connor–Davidson Resilience Scale (CD-RISC) (18). Two versions of the CD-RISC were used in different hospitals. The original version consists of 25 items, each rated on a 5-point scale (0 = not true at all to 4 = true nearly all the time). Total scores range between 0 and 100 points, with higher scores reflecting greater resilience. The 10-item version screened out 10 items based on the original scale, with a total score of 0–40. Existing studies have confirmed that the 10-item short version also has good psychometric properties (19, 20). Cronbach’s alpha was 0.87 for original version and 0.89 for 10-item version in the current study. In the final analysis, resilience scores were calculated using the questions in the 10-item version for both hospital sites.

#### Childhood trauma

The Childhood trauma was measured using Childhood Trauma Questionnaire-short form (CTQ-SF), which consists of 28 items (21), covering 5 types of neglect or abuse (emotional neglect, physical neglect, emotional abuse, physical abuse, and sexual abuse). Each item uses a 5-level score to evaluate the frequency of trauma. The total score is between 25 and 125 points, with higher scores reflecting high level of trauma exposure. The scale has been validated for its reliability and validity in the Chinese schizophrenia population (8). The Cronbach’s alpha in this study was 0.81.

### Statistical analysis

At the outset, descriptive statistics for demographic characteristics of the sample were computed using Stata software, version 16.0 for Mac. Continuous variables are presented as the means and standard deviation. Categorical variables are presented as counts and percentages. Latent profile analysis (LPA) was used to identify the potential classes of resilience. A series including Akaike information criterion (AIC), sample size-adjusted Bayesian information criterion (saBIC), entropy and bootstrapped likelihood ratio test (BLRT) were chosen to identify the optimal solution of LPA analysis. A generalized additive mixed model (GAMM) and restricted cubic spline (RCS) were used to explore the potential non-linear relationship between resilience and childhood trauma. The GAMM model is an extension of the generalized additive model (GAM) model, which contains a link function and smoothed function to deal with high non-linear relationships (22–24). RCS fitted for ordinary least squares (OLS) regression models determined the shape of the relationship between continuous resilience and childhood trauma without any *a priori* assumption of linearity (25). These analyses were performed using R software, version 4.2.0. Three packages, including “tidyLPA” (version 1.1.0), “mgcv” (version 1.8-40) and “rms” (version 6.3-0), were used for LPA, GAMM and RCS analysis, respectively. The level of significance was set to 0.05 (2-tailed).

## Results

Six hundred thirty-six participants with diagnoses of schizophrenia were included in the study. Three hundred forty participants were measured based on the original CD-RISC scale, and two hundred ninety-six participants were measured based on the 10-item version. The demographic variables and clinical characteristics of the subjects are shown in Table 1. The mean age of our participants was 37.09, and more than half of the sample was male and single. The majority of our sample had less than a high school education (69.34%). The results of univariate regression analysis showed that age, marital status, education and family history had no statistically significant effect on resilience.

**TABLE 1:** Basic demographics of the study sample and the univariate relationship with resilience.

Variables	N/mean	%/SD	Coefficients	*t*-value	*p*-value
Age (mean/SD)	37.09	9.87	0.004	0.15	0.822
**Sex (N/%)**					
Men	379	59.59%	–	–	–
Women	257	40.41%	−0.18	−0.38	0.706
**Marriage status (N/%)**					
Alone	381	59.91%	–	–	–
Married	134	21.07%	0.30	0.47	0.640
Divorce	116	18.24%	0.43	0.67	0.500
Widowed	5	0.79%	−2.28	−1.29	0.196
**Education (N/%)**					
Primary and less	267	41.98%	–	–	–
Junior high school	174	27.36%	0.63	1.12	0.263
High school	115	18.08%	0.85	1.26	0.208
University and above	80	12.58%	1.59	1.86	0.063
**Family history (N/%)**					
No	319	50.24%	–	–	–
Yes	316	49.76%	0.78	1.63	0.104

SD, standard deviation.

The model fits of LPA are shown in Table 2. The AIC and sample size-adjusted BIC did not reach a minimum, and the BLRT was significant for each successive model. The entropy, as the index of classification accuracy of model fit, decreased with increasing model complexity. The model fit index does not explicitly point to model selection schemes. In the model selection, the interpretability and stability of the model should also be considered, and the proportion of the number of people in each group should be at least 5% of the total sample (26–28). Based on the consideration above, a three-profile solution was accepted in our study.

**TABLE 2:** Model fit statistics for the 1- to 5-profile solutions.

Class	AIC	saBIC	Entropy	BLRT_p	N_min	N_max
1	17009	17035	1.00		1.00	
2	15750	15790	0.808	0.01	0.487	0.513
**3**	**15464**	**15517**	**0.780**	**0.01**	**0.241**	**0.491**
4	15399	15467	0.779	0.01	0.047	0.442
5	15382	15464	0.735	0.01	0.066	0.308

AIC, Akaike information criterion; saBIC, sample adjusted Bayesian information criterion; BLRT, bootstrapped likelihood ratio test. Bold typeface indicates the best model.

**FIGURE 1:**
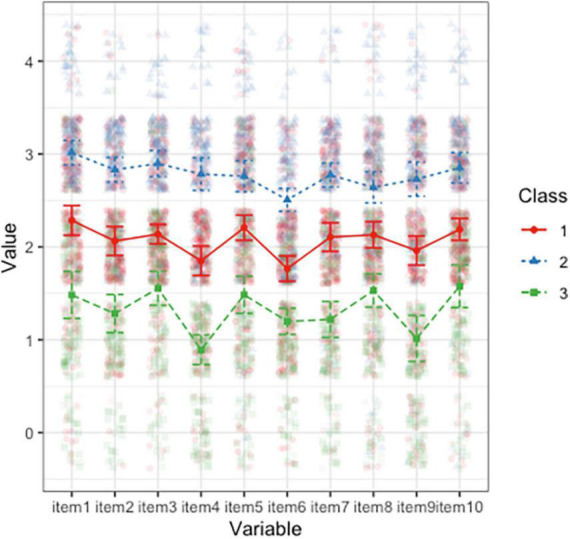
The three-profile model based on the performance of 10 items of the Connor–Davidson Resilience Scale (CD-RISC).

Figure 1 shows the three-profile model following LPA. Members of the first profile reported moderate resilience levels (*n* = 312; 49.06%). Members of the second profile (*n* = 171; 26.89%) had high levels of resilience. Members of the third profile (*n* = 153; 24.06%) exhibited low levels of resilience. The sample characteristics of each profile are presented in Table 3. Statistical analysis showed that there was no significant difference in age, marital status, education level, or family history among the three groups. As the data show, the three groups of samples have similar ranges in childhood trauma levels, while the resilience levels show differences, with resilience scores of 20.68, 28.05, and 12.96 for Group 1, Group 2, and Group 3, respectively, which suggests that there may be a complex non-linear relationship between resilience and childhood trauma.

**TABLE 3:** Basic demographic and clinical characteristics of the three classes.

	Class 1 (*n* = 312)	Class 2 (*n* = 171)	Class 3 (*n* = 153)	Difference
Age (mean/SD)	37.56 (9.63)	36.64 (9.48)	36.61 (10.73)	0.276^a^
Sex (N/%)				0.591^b^
Men	129 (61.54%)	100 (58.48%)	87 (56.86%)	
Women	120 (38.46%)	71 (41.52%)	66 (43.14%)	
Marriage status (N/%)				0.812^c^
Alone	185 (59.29%)	100 (58.48%)	96 (62.75%)	
Married	66 (21.15%)	37 (21.64%)	31 (20.26%)	
Divorce	58 (18.59%)	34 (19.88%)	24 (15.69%)	
Widowed	3 (0.96%)	0	2 (1.31%)	
Education (N/%)				0.268^b^
Primary and less	131 (41.99%)	62 (36.26%)	74 (48.37%)	
Junior high school	90 (28.85%)	47 (27.49%)	37 (24.18%)	
High school	57 (18.27%)	33 (19.30%)	25 (16.34%)	
University and above	34 (10.90%)	29 (16.96%)	17 (11.11%)	
Family history (N/%)				0.237^b^
No	151 (48.40%)	82 (48.24%)	86 (56.21%)	
Yes	161 (51.60%)	88 (51.76%)	67 (43.79%)	
Resilience (mean/SD)	20.68 (2.38)	28.05 (2.91)	12.96 (2.99)	<0.001^a^
CTQ scores (mean/SD)	45.28 (9.51)	45.16 (11.84)	45.66 (10.01)	0.753^a^

SD, standard deviation; ^a^analysis of variance; ^b^chi-square test; ^c^Fisher’s precision probability test.

The abscissa represents the 10 items of the 10-item version of the CD-RISC, and the ordinate represents the standardized mean resilience score.

To further analyze the relationship between childhood trauma and resilience, using resilience as a continuous variable, a generalized additive mixed model (GAMM) was performed to explore the potential non-linear relationship between resilience and childhood trauma. The GAMM results verified the non-linear relationship between resilience and childhood trauma, with an estimated degree of freedom higher than 1 (4.573) and *p*-value < 0.05 (F-statistic 3.936, *p* < 0.003) (22).

Based on the results of the GAMM, the RCS was further employed to delineate the curvilinear shape between childhood trauma and resilience. According to the suggestion by Harrell (25), 4 knots were most often used, which can take into account the smoothness of the curve and avoid the reduction of accuracy caused by overfitting. At the same time, the RCS splits by three to five knots, and AIC was chosen to determine the best knots in our study (29, 30). The AIC for 3, 4, and 5 knots was 4086.343, 4074.773, and 4076.985, respectively, which supports 4 knots as the best solution.

**FIGURE 2:**
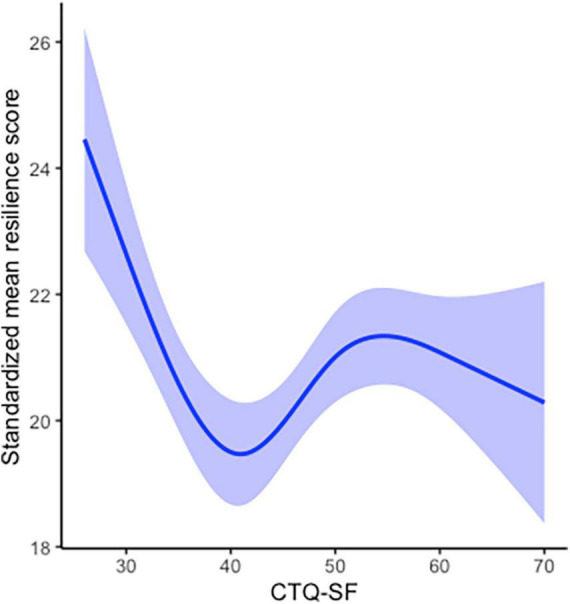
Restricted cubic spline plot demonstrating the association between resilience and childhood trauma. The solid line represents the optimal fitting curve. The shadow indicates the 95% confidence intervals.

Figure 2 shows the RCS fitted for OLS regression models that determined the cubic curve shape of the relationship between resilience and childhood trauma. The results of model fit showed that the two turning points of the curve line were 41.04 and 54.74, which indicated that when the trauma score was lower than 41.04 or higher than 54.74, the resilience score decreased with increasing trauma score. When the trauma score was in the range of 41.04–54.74, the resilience score increased with increasing trauma score.

## Discussion

The study of resilience has challenged the long-standing view of the negative impact of traumatic events on individuals, and researchers have begun to focus on the positive impact of traumatic experiences on individual psychological development from a positive perspective. In the field of schizophrenia research, there has been limited research that has directly focused on people-specific differences in resilience and its non-linear relationship to childhood trauma. Based on the resources we reviewed, this study is the first to identify the heterogeneity of resilience based on latent profile analysis and to further reveal a curvilinear relationship between resilience and childhood trauma among people with schizophrenia.

One of our findings is that there are three heterogeneous subgroups of resilience among people with schizophrenia. Zhang et al. (31) used CD-RISC to analyze the structural characteristics of resilience of Chinese empty-nest elderly people, and the results identified three heterogeneous subgroups: “high resilience group,” “low pressure resilience group,” and “low resilience group.” Age, sex, marital status, and education level had significant effects on subgroup characteristics. Three similar groups of structural characteristics of resilience have been verified in different populations and scenarios (32–34). Although there are differences in the population characteristics, scale tools and methods used in different studies, the three subgroup structural characteristics of resilience are relatively stable, which is consistent with the results of this study. However, different heterogeneous solutions of resilience were also found in other populations (35), which may be due to differences in samples and needs to be further verified and summarized. However, as a people-centered statistical analysis method, the purpose of subgroup identification is to accurately identify and characterize population characteristics. However, no significant differences were found between subgroups in terms of age, gender, marital status and education level in this study. Subsequent studies need to further incorporate more characteristics and explore the specific factors of subgroups to achieve the prediction of subgroups based on different characteristics.

Another finding of this study is that there is a cubic curvilinear relationship between childhood trauma and resilience. When there may be non-linear relationships between variables, continuous variables are often divided into categorical variables, or piecewise regression is performed, but this processing method often leads to a lack of information (25). To achieve an accurate description of the relationship between variables as much as possible, our study used RCS to fit a smooth continuous curve of resilience across childhood trauma levels, which showed a cubic curvilinear relationship and two turning points between childhood trauma and resilience. The “steeling effect” showed that there is a moderate turning point in the effect of traumatic events on the individual, so that the impact of trauma on the individual becomes a positive tendency to improve the individual’s ability to adapt and cope (13, 36, 37). Our results are consistent with the “steeling effect” of childhood trauma for resilience, which showed two nodes that alter the effects of trauma on resilience. The “challenge model” further characterizes the relationship between risk factor exposure and resilience. The “challenge model” of resilience states that the relationship between risk factors and resilience is curvilinear, and only exposure to moderate levels of risk factors can help individuals acquire the ability to adapt and deal with subsequent stress. It emphasizes that exposure to risk factors must be sufficient, but not excessive, and exposure levels that are too low or too high are not beneficial to individuals (38–40). Our findings confirm the “challenge model” of resilience in schizophrenia, which are consistent with previous study (41). When individuals with schizophrenia are exposed to lower levels of childhood trauma, they cannot obtain sufficient “inoculation effects,” and as the level of trauma increases, the level of individual resilience decreases. When the trauma level is too high, it will destroy the individual’s coping system, and resilience will also decrease with the increase in the trauma level. There is a positive effect between resilience and trauma level only when individuals are in a moderate trauma exposure range.

Resilience plays an important role in the full course management of schizophrenia, and in fully recovered patients, resilience promotes the positive value of recovery (6, 7, 42). Enhancing and improving the resilience level of the schizophrenia population is important. The existing research on the verification of challenge model is very limited. This study is the first to explore the potential heterogeneity class of resilience and validate the “challenge model” of childhood trauma on resilience in schizophrenia. The results showed that detailed characterization of resilience in schizophrenia based on different subgroups is needed, and only moderate stress experience can activate the inoculation effect and produce benefits. Future studies should continue to clarify the heterogeneous structural features and series of biomarkers of resilience, so as to provide reference for relevant intervention studies.

## Conclusion

Our findings confirm the people-specific differences in the level of resilience in schizophrenia and describe the cubic curvilinear relationship between childhood trauma and resilience. Sufficient but not excessive trauma experience may increase resilience. The results of the study can help guide the development of individualized stress inoculation programs based on differences in subgroup characteristics to enhance their resilience.

## Limitations

Our study has the following limitations. First, our samples are all from cities in northern China, so the representativeness of the conclusions is limited. Second, although our findings confirmed the non-linear relationship between childhood trauma and resilience and divided the interval cutoff value of the “trauma positive effect” based on statistical methods, the division of the optimal cutoff value has been inconsistent among other studies (25, 43), so our results still need further verification. Third, although the results of this study validate the “challenge model” of resilience in schizophrenia, resilience is a complex concept, and the impact of a series of protective factors and their interactions on resilience needs to be further validated. Finally, no healthy control group was included in this study, so the “challenge model” of resilience, namely the cubic curve relationship between trauma and mental resilience, needs to be further verified in other populations.

## Data availability statement

The raw data supporting the conclusions of this article will be made available by the authors, without undue reservation.

## Ethics statement

The studies involving human participants were reviewed and approved by the Medical Ethics Committee of Harbin Medical University (Daqing). Written informed consent to participate in this study was provided by the participants’ legal guardian/next of kin.

## Author contributions

WW and YZ conceptualized and designed the study. YZ and GL supervised the data collection. WW, JZ, and XZ undertook the recruitment of subjects and managed the data. WW drafted the manuscript. YZ obtained the funding and supervised the study. All authors read and approved the final version of the manuscript.
